# Achilles tendon rupture healing is enhanced by intermittent pneumatic compression upregulating collagen type I synthesis

**DOI:** 10.1007/s00167-017-4621-8

**Published:** 2017-07-01

**Authors:** Md. Abdul Alim, Erica Domeij-Arverud, Gunnar Nilsson, Gunnar Edman, Paul W. Ackermann

**Affiliations:** 1 Integrative Orthopedic Laboratory, Department of Molecular Medicine and Surgery, Karolinska Institutet, Stockholm, Sweden; 2 Department of Orthopedics, Karolinska University Hospital, 171 76 Stockholm, Sweden; 3 Department of Psychiatry, Tiohundra AB, Norrtälje, Sweden; 4 Department of Orthopedics, Danderyd Hospital, Stockholm, Sweden

**Keywords:** Achilles tendon rupture, Regeneration, Intermittent pneumatic compression devices, Microdialysis, Procollagen

## Abstract

**Purpose and hypothesis:**

Adjuvant intermittent pneumatic compression (IPC) during leg immobilization following Achilles tendon rupture (ATR) has been shown to reduce the risk of deep venous thrombosis. The purpose of this study was to investigate whether IPC can also promote tendon healing.

**Methods:**

One hundred and fifty patients with surgical repair of acute ATR were post-operatively leg immobilized and prospectively randomized. Patients were allocated for 2 weeks of either adjuvant IPC treatment (*n* = 74) or treatment-as-usual (*n* = 74) in a plaster cast without IPC. The IPC group received 6 h daily bilateral calf IPC applied under an orthosis on the injured side. At 2 weeks post-operatively, tendon healing was assessed using microdialysis followed by enzymatic quantification of tendon callus production, procollagen type I (PINP) and type III (PIIINP) N-terminal propeptide, and total protein content. 14 IPC and 19 cast patients (control group) consented to undergo microdialysis. During weeks 3–6, all subjects were leg-immobilized in an orthosis without IPC. At 3 and 12 months, patient-reported outcome was assessed using reliable questionnaires (ATRS and EQ-5D). At 12 months, functional outcome was measured using the validated heel-rise test.

**Results:**

At 2 weeks post-rupture, the IPC-treated patients exhibited 69% higher levels of PINP in the ruptured Achilles tendon (AT) compared to the control group (*p* = 0.001). Interestingly, the IPC-treated contralateral, intact AT also demonstrated 49% higher concentrations of PINP compared to the non-treated intact AT of the plaster cast group (*p* = 0.002). There were no adverse events observed associated with IPC. At 3 and 12 months, no significant (n.s.) differences between the two treatments were observed using patient-reported and functional outcome measures.

**Conclusions:**

Adjuvant IPC during limb immobilization in patients with ATR seems to effectively enhance the early healing response by upregulation of collagen type I synthesis, without any adverse effects. Whether prolonged IPC application during the whole immobilization period can also lead to improved long-term clinical healing response should be further investigated. The healing process during leg immobilization in patients with Achilles tendon rupture can be improved through adjuvant IPC therapy, which additionally prevents deep venous thrombosis.

**Level of evidence:**

Randomized controlled trial, Level I.

**Electronic supplementary material:**

The online version of this article (doi:10.1007/s00167-017-4621-8) contains supplementary material, which is available to authorized users.

## Introduction

Patients with acute Achilles tendon rupture (ATR) are at high risk of immobilization-induced complications such as deep venous thrombosis (DVT) [8, 9] and a prolonged, impaired healing process with an unpredictable variation in final individual long-term outcome [16]. Although early mobilization is advocated after ATR, the most optimal post-injury care protocol in order to counteract immobilization-induced impairments on the healing process is unknown [12].

Leg immobilization causes reduced blood flow, [6] hampered metabolism and decreased production of collagen type I and III after ATR, which all result in an impaired healing process [4, 19]. Recently however, adjuvant intermittent pneumatic compression (IPC), which increases blood circulation during leg immobilization, was demonstrated to increase metabolism [10] and to reduce the risk of DVT after ATR [8, 9].

Moreover, IPC has experimentally been shown to improve ATR repair by generating enhanced collagen production and thereby improved tensile strength during immobilization [7, 19]. To assess collagen formation in human Achilles tendon microdialysis followed by quantification of procollagen N-terminal propeptide type III (PIIINP) and type I (PINP), which are the major building stones in the tendon, has been used [13]. Whether adjuvant IPC treatment in ATR patients can counteract immobilization-induced impairments, by improving callus production has been unknown.

In this study, it was hypothesized that 2 weeks of calf IPC beneath orthosis immobilization could increase the callus production with regard to PINP and PIIINP compared to treatment-as-usual with plaster cast. A secondary aim was to investigate the long-term functional and patient-reported outcome at 3 and 12 months post-operatively.

Adjuvant IPC therapy could, in addition to prevention of DVT, improve healing outcome of ATR patients.

## Materials and methods

**Fig. 1:**
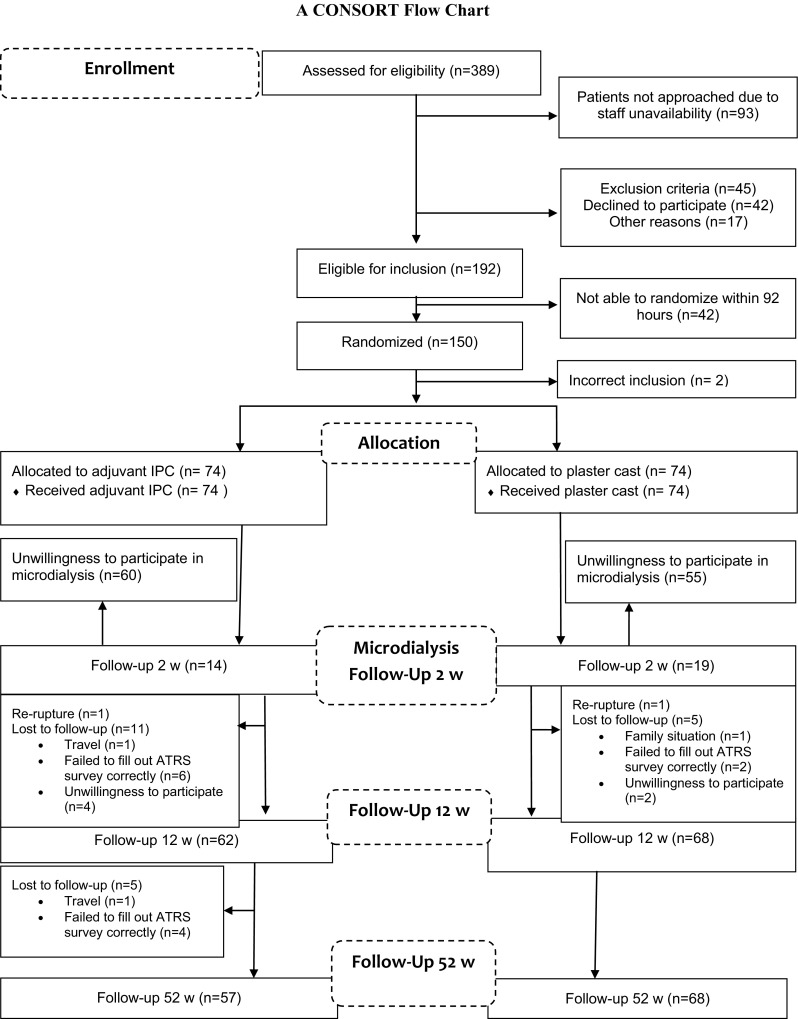
CONSORT study flow diagram. Patients with ATR were randomized to either adjuvant IPC (*n* = 74) or treatment-as-usual in a plaster cast without IPC (*n* = 74) for the first 2 weeks after ATR surgical repair. At 2 weeks, the intervention with IPC stopped, and subsequently all patients were treated with the same protocol for rehabilitation

This prospective randomized control study was conducted in the Outpatient Orthopedic/Sports Medicine Department, Karolinska University Hospital, Solna, and was designed and reported according to the CONSORT (Consolidated Standards of Reporting Trials) guidelines (Fig. 1). Ethical approval was obtained from the Regional Ethical Review Committee in Sweden. All participants received oral and written information about purpose and procedures of the study and provided written informed consent of the study. The trial was registered with the United States National Institutes of Health (trial number NCT01317160). This study was single-blinded, controlled, and applied block randomization.

### Participants, eligibility criteria and randomization

Patients between 18 and 75 years of age with a unilateral ruptured Achilles tendon that had undergone surgery within 96 h of injury were eligible for inclusion. The exclusion criteria were: inability to give informed consent; current anticoagulation treatment (including high dose acetylsalicylic acid); renal failure; heart failure with pitting oedema; thrombophlebitis; thromboembolic event in the previous three months; known malignancy; haemophilia; pregnancy; other surgery during the previous month; inability to follow instructions and planned follow-up at another hospital.

Between 2011 and 2013, 389 patients with ATR were screened for eligibility (Fig. 1). One hundred and fifty patients (126 men and 24 women) with a mean age of 40 years (range 18–71) were included and underwent Achilles tendon repair. Patients were enrolled to the interventions either by a third party nurse or by a research nurse. Randomization was performed with use of computer-generated random numbers in permuted blocks of four, through an independent software specialist, and consecutively numbered, sealed, opaque envelopes opened after surgery and prior to treatment. The patients were randomized to receive either standard plaster cast treatment alone or calf IPC beneath an orthotic device.

Two patients were initially included in error, one in each group: one had pre-existing DVT and one was below 18 years of age. They were both withdrawn from the study immediately after randomization. For the analysis with microdialysis and quantification of procollagen levels at 2 weeks, the treatment group comprised 14 patients (IPC beneath an orthosis) and the control group contained 19 patients (cast alone), which was an adequate number as calculated in the power analysis.

### Achilles tendon suturing technique and post-operative treatment protocol

#### Allocation and surgical procedure

Twenty-three specialists and 15 resident orthopaedic surgeons at one university hospital were randomly allocated to operate on each patient according to the shift duty schedule. The surgical procedure was performed on an outpatient basis using local anaesthesia and modified Kessler suture technique as described elsewhere [8]. The surgical techniques were standardized for suturing technique and suture type.

#### IPC group

Patients in the IPC group received 2 weeks of bilateral calf IPC (Venaflow^®^ Elite, DJO LLC, Vista, CA, USA) applied on the calf of the injured limb under an orthosis with 3 heel wedges (Aircast^®^ XP Walker™, DJO LLC, Vista, CA, USA) [8]. Patients were allowed to weight-bear as tolerated.

Weight-bearing was registered by daily patient-reported loading. Each patient was asked to record the loading on the injured limb as a percentage of full loading in a subjective way, for the first 2 weeks of the rehabilitation period. Additionally, the number of steps was recorded daily by a pedometer (Keep Walking’ LS 2000/SW 200) attached to the walker boot. The aim was to analyse daily loading in relation to callus production.

The patients exhibited low self-reported loading (day 5: mean load 33 ± 25%) and low number of steps (day 5: 836 ± 1069) during the first week. The second week patients exhibited successive increased loading (day 13: mean load 69 ± 29%) and increased number of steps (day 13: 1950 ± 2041). Neither self-reported loading nor the number of steps were significantly correlated with any of the callus markers analysed.

The patients were instructed to apply the IPC therapy during the time they were sedentary, i.e. sitting or lying in bed sleeping, 6 h at a minimum daily. Patient compliance was registered by both the patient and the device. IPC intervention was discontinued at 2 weeks post-operatively. During weeks 3–6 patients were immobilized in an orthosis (Aircast^®^ XP Walker™, DJO LLC, Vista, CA, USA) and were instructed to bear full weight.

#### Control group

Shortly after the completion of surgery, the patients of the control group received treatment-as-usual, i.e. a below-knee plaster cast with the ankle in 30 degrees equinus position in the outpatient clinic and were non-weight-bearing with crutches during the first 2 weeks. During weeks 2–6, patients were immobilized, similar to the IPC group, in an orthosis (Aircast^®^ XP Walker™, DJO LLC, Vista, CA, USA) and were instructed full weight-bearing.

At 6 weeks post-operatively, all patients, from both study groups, were discontinued orthosis immobilization.

### Patient characteristics and assessments

#### Patient characteristics

The patients exhibited no significant differences in age, sex, smoking habits and BMI between the IPC and the control groups (Tables 1, 2).

**Table 1:** Patient characteristics at 2-week microdialysis follow-up

	Plaster cast group	IPC group	*p*
Age* (years)	40 ± 7.5	41 ± 8	n.s
Sex (M/F)	18 (95%)/1 (5%)	12 (86%)/2 (14%)	n.s
Height* (cm)	179 ± 7.5	179 ± 8.4	n.s
Weight* (kg)	84.5 ± 11	86.6 ± 12.7	n.s
BMI*	26.2 ± 2.7	27.1 ± 3.6	n.s
Smoker, yes/no	1 (5%)/18 (95%)	1 (7%)/13 (93%)	n.s

*M/F* male and female, *BMI* Body Mass Index; *P* (*n.s*) non-significance *p* value

* The values are denoted as the Mean ± standard deviation

**Table 2:** Patient characteristics at 1-year follow-up

	Plaster cast group	IPC group	*p*
Age* (years)	40 ± 10	41 ± 8	n.s
Sex (M/F)	63 (85%)/11 (15%)	49 (91%)/5 (9%)	n.s
Height* (cm)	178 ± 8	179 ± 8	n.s
Weight* (kg)	84 ± 13	87 ± 13	n.s
BMI*	26 ± 3	27 ± 4	n.s
Smoker, yes/no	4 (6%)/69 (94%)	0/54 (100%)	n.s

*M/F* male and female, *BMI* body mass index, *p* (*n.s*) non-significance *p* value

* The values are denoted as the Mean ± standard deviation

The IPC-treated patients exhibited a mean of 71.3 h (range 18.1–178.5) in the self-registered application of IPC, which correlated well with the device registered usage of 71.3 h (range 40.0–178.5).

#### Microdialysis

In order to assess tendon healing, microdialysis followed by procollagen and protein analyses was performed after insertion of a catheter at the volar aspect of the paratenon as described earlier [10], and as demonstrated in the attached video (see Video S1, Supplemental Digital Content 1). Microdialysis was conducted at the 2-week post-operative control by an examiner who was blinded to the intervention.

#### Quantification of procollagen types I and III and of protein content

In order to assess markers of callus production, the N-terminal propeptides of procollagen I (PINP) and procollagen III (PIIINP) as well as the total protein content were quantified in the microdialysate [2]. The PINP (Catalog no # SEA957Hu) and PIIINP (Catalog no # SEA573Hu) levels were measured via a sandwich enzyme-linked immunosorbent assay (ELISA) kit as per manufacturer’s instructions (USCN Life Science, Inc., Houston, TX, USA) and the total protein content was assessed with the Bradford protein assay. The qualitative, normalized procollagen levels, n-PINP and n-PIIINP, were calculated by dividing the concentrations of PINP and PIIINP, respectively, by the total protein content.

#### Patient-reported outcome and physical activity

The patients’ symptoms and physical activity level were assessed using four reliable and valid scores, the Achilles tendon Total Rupture Score (ATRS, Swedish, version 6;) [15, 23], Physical Activity Scale (PAS) [11], Foot and Ankle Outcome Score (FAOS Swedish version LK 1.0) [18] and EuroQol Group’s questionnaire (EQ-5D) [5].

#### Functional outcome

Muscular endurance testing was conducted at 12 months post-operatively on both limbs as has previously been described [20, 21] using MuscleLab (Ergotest Technology Oslo, Norway). The tests have been shown to be reliable and valid and have frequently been used to evaluate the outcome after ATR [14, 16, 21, 22]. The Limb Symmetry Index (LSI = (injured/uninjured) × 100) was calculated for heel-rise height, repetitions and total work.

The IRB approval was permitted from the Ethical Review Board, Stockholm, with ID number 2009/2079–31/2.

### Statistical analysis

The sample size for the tendon callus production was calculated on a difference of the PINP concentration of 1000 pg/ml between the two groups. It was determined that a sample size of 13 patients per group would be necessary to detect the glutamate difference with 80% power when alpha was set equal to 5%. The sample size for the patient-reported outcome was calculated on a clinically relevant difference of ATRS at 1 year of 10 points and the standard deviation was estimated to be 15 points on the basis of an earlier study [7]. Thirty-six patients were needed in each group and we included 70 patients to account for loss at 1-year follow-up and subgroup analyses.

All variables were summarized with standard descriptive statistics including mean, standard deviation (SD), and frequency. All statistics, descriptive as well as analytic, were calculated using SPSS (v22.0, Armonk, NY, USA). All variables were checked for severe skewness.

Differences between the IPC and control group were analysed with the nonparametric Mann–Whitney *U* test for skewed data and with the independent samples *t* test for normally distributed data. Differences in levels of callus markers between the injured and intact legs were analysed with Wilcoxon matched-pairs test. Correlations between weight-bearing (loading and number of steps) and callus formation parameters were performed with Pearson and Spearman analytic tests for parametric and nonparametric values, respectively. The level of significance was set to ≤0.05 (two-tailed).

## Results

### Callus production in the IPC versus plaster cast group

**Fig. 2:**
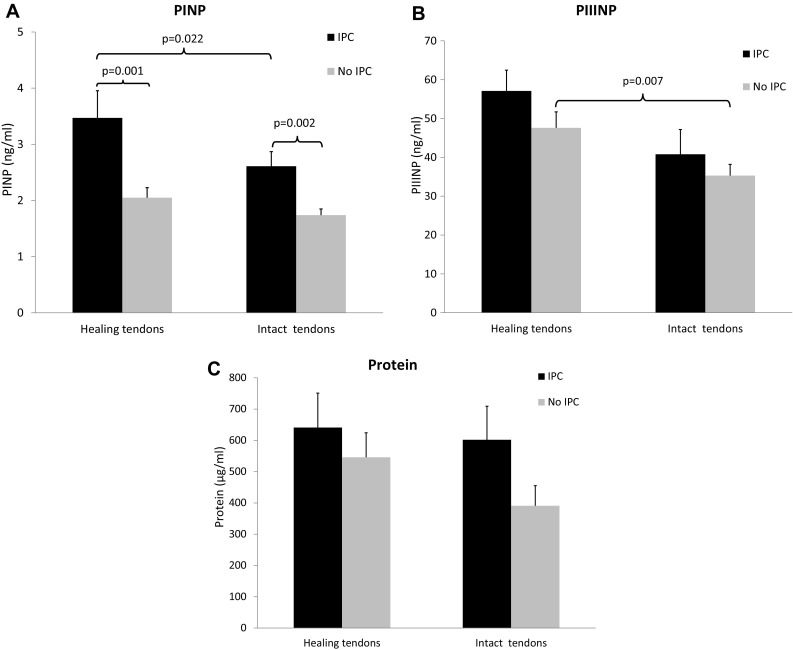
Callus production without and with IPC. Concentration of markers of callus production, **a** procollagen type I (PINP), **b** procollagen type III (PIIINP), and **c** total protein content, in the IPC-treated tendons versus the tendons without IPC treatment. Callus production is assessed in both the healing, i.e. ruptured Achilles tendons (*n* = 14 IPC, *n* = 19 no IPC) and the contralateral intact Achilles tendons (*n* = 14 IPC, *n* = 18 no IPC). Data are expressed as mean ± SEM. ** *p* < 0.01, *** *p* < 0.0001

Patients in the IPC-treated group exhibited 69% higher concentrations of PINP (*p* = 0.001) in their healing AT as compared to the plaster treated group (Fig. 2a). PIIINP and protein concentrations were not significantly different between groups (Fig. 2b, c).

Notably, the IPC-treated intact AT also exhibited 49% higher concentrations of PINP compared to the non-treated intact AT of the plaster cast group (*p* = 0.002; Fig. 2a). The other markers of callus production did not display any significant differences between the intact AT of the two groups (Fig. 2b, c).

Also when comparing the healing versus intact contralateral AT, patients in the IPC group exhibited significantly elevated callus concentrations (Fig. 2a). Hence, the healing compared to intact AT displayed 33% higher levels of PINP (*p* = 0.022) in the IPC group. The plaster cast group, on the contrary, exhibited higher levels of PIIINP in the healing AT compared to the contralateral, intact tendons (*p* = 0.007; Fig. 2b). No other significant differences were found between the healing versus intact AT (Fig. 2c).

### Patient-reported outcome in the IPC versus plaster cast group

No significant differences between the two treatment groups were observed in patient-reported outcome measures (ATRS, FAOS, PAS and EQ-5D), neither at three nor at twelve months (Table S2 & S3, Supplemental Digital Content 2–3). ATRS was at 3 months 46.3 ± 16.7 and 43.2 ± 18.5, in the IPC- and plaster groups, respectively. At 1 year, ATRS increased evenly in the IPC 77.8 ± 17.2 and plaster cast groups 78.0 ± 19.3.

### Functional outcomes in the IPC versus plaster cast groups

Assessments at 1 year post-operatively with the functional heel-rise tests did not display any significant differences between the IPC- and plaster cast treated groups in any of the outcome measures (see Table S4, Supplemental Digital Content 4). Limb Symmetry Index (injured/uninjured) × 100 of average heel-rise height was 82.5 ± 17.0% in the IPC group and 78.5 ± 14.6% in the plaster cast group (n.s.).

### Adverse events

There were no adverse events observed associated with IPC. On the contrary, at 2 weeks the rate of deep venous thrombosis was 21% in the IPC group and 38% in the control group (OR = 2.36; 95% CI 1.11–5.01) [8]. At 6 weeks post-operatively, there were two local wound infections in the IPC group (3.0%) and four in the control group (5.5%). Two patients with re-ruptures were observed, one from each group.

## Discussion

This prospective randomized study demonstrated that 2 weeks of adjuvant IPC therapy during lower limb immobilization in patients with ATR significantly upregulated the early healing response as assessed by quantification of markers of callus production at 2 weeks post-operatively. Moreover, no long-term adverse effects of IPC were observed on the patient-reported and functional outcomes at 1 year.

The innovative step in this study involved the usage of IPC therapy under leg immobilization in an orthosis, which led to elevated production of procollagen in the treatment group. Earlier studies examined the incidence of DVT at 2 weeks post-operatively as primary outcome [8, 9]. Adjuvant IPC was shown to be an effective method to reduce the risk of DVT during leg immobilization of ATR patients. Here we demonstrated that IPC treatment during limb immobilization in addition to lowering the risk of DVT seems to improve the early healing as reflected by higher procollagen levels.

The most important finding of the present study pertained to the around 70% upregulation of PINP in the healing tendons of the IPC-treated compared to the plaster cast group. These observations suggest an enhanced repair response in the IPC group, since PINP is a marker of production of collagen type I, which is the strongest and most predominant building stone in the Achilles tendon. The above conclusion was corroborated by recent experimental studies demonstrating that IPC treatment during 2 weeks of leg immobilization after ATR in rats counteracted both biomechanical and morphological deficits caused by an orthosis limb immobilization [19].

The finding that the healing at 2 weeks compared to the contralateral, intact AT of the plaster cast group did not exhibit any upregulation of PINP, suggests that leg immobilization may inhibit collagen type I production. Thus, earlier studies have clearly demonstrated that 2 weeks of leg immobilization impairs proliferative repair after ATR with up to 80% loss in tensile strength attributed to abolished collagen type I production [4, 19]. Therefore, the observed elevated levels of PINP in the healing IPC-treated compared to both the contralateral intact AT and the plaster casted healing AT suggests that compression treatment is an effective therapy to counteract impairments of the healing process associated with leg immobilization.

The non-significant elevation of PIIINP in the healing tendons of the IPC- compared with the plaster immobilized group presumably reflects that the IPC group has entered a more progressed healing stage as compared to the plaster cast group. An elevation of PIIINP would indicate synthesis of collagen type III, which is the unorganized, “scar” collagen produced in the initial phase, in the first 2 weeks of healing. While the observed increase in the IPC group of PINP, which reflects synthesis of the strong, high-quality and organized collagen type I usually seen after 2 weeks of healing, would implicate a progressed healing stage. The finding that the healing compared to intact AT of the plaster cast group exhibited an increase of PIIINP, which was not observed in the IPC group would seem to corroborate the above conception.

Hence, the finding of upregulated PINP in the healing AT of the IPC group is presumably reflective of a whole cascade of activated proliferative healing mechanisms. Thus, IPC has in addition to enhancement of both venous and arterial blood flow during ATR healing been demonstrated to promote neurovascular ingrowth, fibroblast proliferation, and to increase the supply of sensory neuropeptides to the healing connective tissue [4, 19].

Recently, it was furthermore demonstrated that in the early stage of human ATR healing there is a scarce microcirculation [3] and low metabolism, which can be increased by IPC [10]. Hence, local essential metabolites are upregulated during ATR healing and these levels can be further enhanced by IPC during plaster cast immobilization [10].

The second main finding of this study pertained to the observation of elevated PINP levels also in the intact Achilles tendons of the IPC versus plaster cast group. This finding indicates that the proliferative effects of the compression treatment also can be applied to intact tendon metabolism. Moreover, this result also suggests that weight-bearing did not affect the increased PINP levels since the intact weight-bearing AT of the plaster cast group may be expected to exhibit higher load than the intact AT of the IPC group. This observation was strengthened by the finding that the patient-reported loading was not correlated with the levels of PINP.

The observed healing stimulatory effects of IPC during immobilization as seen in ATR can presumably also be applied to other soft tissue or bone injuries in need of immobilization. In fact, women with low bone mass [1] and experimental fractures [17] have demonstrated improved mineralization and torsional strength after compression treatment and moreover different types of connective tissue injuries have demonstrated enhanced healing after IPC [7, 17, 19].

A potential limitation of this study is that we cannot conclude the exact time length that the enhanced healing response associated with adjuvant IPC therapy will persist. The observations of equal patient-reported- and functional outcomes between the two groups at three and twelve months post-operatively demonstrated that the 2 weeks IPC intervention did not improve outcome measures from three months onwards.

However, after the end of the IPC intervention at week two, both treatment groups received immobilization in an orthosis until 6 weeks post-operatively when immobilization was ended. This suggests that the healing stimulatory effects of the IPC therapy do not persist after cessation of treatment when continued immobilization is applied. This conclusion warrants additional studies where the IPC therapy should be applied during the whole time of post-operative lower limb immobilization. By applying IPC treatment during 6 weeks the therapy would impact both the proliferative as well as the regenerative healing phases during immobilization, which could conceivably affect also the patient-reported and functional outcomes as well as lead to earlier return to activity.

Whether mechanical compression therapy should be administered as an outpatient treatment for leg-immobilized patients is, with the present and another published study in mind, a matter of both preventing the development of DVT [8, 9] as well as of counteracting the impaired healing associated with immobilization. As for yet no cost–benefit analysis has been performed, yet the therapy is highly accepted by the patients. Further investigation of the health economics of IPC intervention ought to be conducted to permit an informed decision on implementation at a population level.

## Conclusions

In conclusion, patients in post-operative lower limb immobilization after ATR demonstrate a significantly enhanced early healing response when using adjuvant calf IPC for 2 weeks. This study concludes that IPC in addition to exert a prophylactic effect against DVT also may be a viable and effective treatment to prevent immobilization-induced impairments on the healing process. Further studies should assess if prolonged IPC usage during the whole immobilization time could shorten the time to recovery and optimize final outcome.

## Electronic supplementary material

Below is the link to the electronic supplementary material.

Supplementary material 1 (PDF 201 kb)

Supplementary material 2 (MP4 49563 kb)

## Abbreviations

IPC: Intermittent pneumatic compression
ADL: Activity of daily living
AT: Achilles tendon
ATR: Achilles tendon rupture
ATRS: Achilles tendon Total Rupture Score
BMI: Body mass index
CONSORT: Consolidated Standards of Reporting Trials
DVT: Deep venous thrombosis
ELISA: Enzyme-linked immunosorbent assay
EQ-5D: EuroQol Group’s Questionnaire
FAOS: Foot and Ankle Outcome Score
M: Mean
PAS: Physical Activity Scale
PINP: Procollagen type I N-terminal propeptide
PIIINP: Procollagen type III N-terminal propeptide
SD: Standard deviation
SPSS: Statistical Package for the Social Sciences

## References

[1] Albertazzi P, Steel SA, Bottazzi M (2005). Effect of intermittent compression therapy on bone mineral density in women with low bone mass. Bone.

[2] Alim MA, Svedman S, Edman G, Ackermann PW (2016). Procollagen markers in microdialysate can predict patient outcome after Achilles tendon rupture. BMJ Open Sport Exerc Med.

[3] Arverud ED, Persson-Lindell O, Sundquist F, Labruto F, Edman G, Ackermann PW (2016). Microcirculation in healing and healthy Achilles tendon assessed with invasive laser doppler flowmetry. Muscles Ligaments Tendons J.

[4] Bring DK, Reno C, Renstrom P, Salo P, Hart DA, Ackermann PW (2009). Joint immobilization reduces the expression of sensory neuropeptide receptors and impairs healing after tendon rupture in a rat model. J Orthop Res.

[5] Brooks R (1996). EuroQol: the current state of play. Health Policy.

[6] Craik JD, Clark A, Hendry J, Sott AH, Hamilton PD (2015). The effect of ankle joint immobilization on lower limb venous flow. Foot Ankle Int.

[7] Dahl J, Li J, Bring DK, Renstrom P, Ackermann PW (2007). Intermittent pneumatic compression enhances neurovascular ingrowth and tissue proliferation during connective tissue healing: a study in the rat. J Orthop Res.

[8] Domeij-Arverud E, Labruto F, Latifi A, Nilsson G, Edman G, Ackermann PW (2015). Intermittent pneumatic compression reduces the risk of deep vein thrombosis during post-operative lower limb immobilisation: a prospective randomised trial of acute ruptures of the Achilles tendon. Bone Joint J.

[9] Domeij-Arverud E, Latifi A, Labruto F, Nilsson G, Ackermann PW (2013). Can foot compression under a plaster cast prevent deep-vein thrombosis during lower limb immobilisation?. Bone Joint J.

[10] Greve K, Domeij-Arverud E, Labruto F, Edman G, Bring D, Nilsson G, Ackermann PW (2012). Metabolic activity in early tendon repair can be enhanced by intermittent pneumatic compression. Scand J Med Sci Sports.

[11] Grimby G (1988). Physical-activity and effects of muscle training in the elderly. Ann Clin Res.

[12] McCormack R, Bovard J (2015). Early functional rehabilitation or cast immobilisation for the postoperative management of acute Achilles tendon rupture? A systematic review and meta-analysis of randomised controlled trials. Br J Sports Med.

[13] Moerch L, Pingel J, Boesen M, Kjaer M, Langberg H (2013). The effect of acute exercise on collagen turnover in human tendons: influence of prior immobilization period. Eur J Appl Physiol.

[14] Nilsson-Helander K, Silbernagel KG, Thomee R, Faxen E, Olsson N, Eriksson BI, Karlsson J (2010). Acute Achilles tendon rupture a randomized, controlled study comparing surgical and nonsurgical treatments using validated outcome measures. Am J Sports Med.

[15] Nilsson-Helander K, Thomee R, Silbernagel KG, Thomee P, Faxen E, Eriksson BI, Karlsson J (2011). The Achilles tendon Total Rupture Score (ATRS): development and validation (vol 35, pg 421, 2007). Am J Sports Med.

[16] Olsson N, Petzold M, Brorsson A, Karlsson J, Eriksson BI, Silbernagel KG (2014). Predictors of clinical outcome after acute Achilles tendon ruptures. Am J Sports Med.

[17] Park SH, Silva M (2003). Effect of intermittent pneumatic soft-tissue compression on fracture-healing in an animal model. J Bone Joint Surg Am.

[18] Roos EM, Brandsson S, Karlsson J (2001). Validation of the foot and ankle outcome score for ankle ligament reconstruction. Foot Ankle Int.

[19] Schizas N, Li J, Andersson T, Fahlgren A, Aspenberg P, Ahmed M, Ackermann PW (2010). Compression therapy promotes proliferative repair during rat Achilles tendon immobilization. J Orthop Res.

[20] Silbernagel KG, Gustavsson A, Thomee R, Karlsson J (2006). Evaluation of lower leg function in patients with Achilles tendinopathy. Knee Surg Sports Traumatol Arthrosc.

[21] Silbernagel KG, Nilsson-Helander K, Thomee R, Eriksson BI, Karlsson J (2010). A new measurement of heel-rise endurance with the ability to detect functional deficits in patients with Achilles tendon rupture. Knee Surg Sports Traumatol Arthrosc.

[22] Silbernagel KG, Thomee R, Eriksson BI, Karlsson J (2007). Continued sports activity, using a pain-monitoring model, during rehabilitation in patients with Achilles tendinopathy—a randomized controlled study. Am J Sports Med.

[23] Spennacchio P, Vascellari A, Cucchi D, Canata GL, Randelli P (2016). Outcome evaluation after Achilles tendon ruptures. A review of the literature. Joints.

