# Supplementary material for: Achilles tendon rupture healing is enhanced by intermittent pneumatic compression upregulating collagen type I synthesis
Source: Knee Surg Sports Traumatol Arthrosc. 2017 Jul 1;26(7):2021–9. doi: 10.1007/s00167-017-4621-8 (PMC6061441; doi:10.1007/s00167-017-4621-8)
Supplement: Supplementary file 1 — Supplementary material 1 (PDF 201 kb) [file 167_2017_4621_MOESM1_ESM.pdf]

Supplemental Digital Content 2.

Table S2. Patient-reported outcome measures in the IPC- vs plaster cast groups at 3 months

**ATRS 3 months**	**IPC**			**Plaster cast**			
	n	M	SD	n	M	SD	p
Strength	62	4.47	2.09	68	4.06	2.34	0.297
Tiredness	61	5.16	2.60	68	4.93	2.59	0.605
Stiffness	62	4.87	2.61	68	5.07	2.59	0.658
Pain	62	7.00	2.56	68	6.97	2.72	0.950
ADL	62	6.00	2.62	68	5.76	2.74	0.619
Uneven surface	60	5.43	2.58	66	5.27	2.64	0.731
Stairs/hills	60	5.12	2.69	66	4.48	2.87	0.206
When running	56	1.36	2.18	66	1.45	2.21	0.808
When jumping	57	1.54	2.39	65	1.23	1.93	0.425
Physical work	59	4.19	3.06	65	3.82	2.69	0.473
**Total sum**	55	46.3	16.7	64	43.2	18.5	0.344

ATRS, Achilles Tendon Ruptured Score; The ATRS consists of 10 questions that can be scored from 0 to 10, leading to a total summed score of 0-100; a lower score indicates more symptoms and greater limitation of physical activity and quality of life; ADL, Activity of daily living; n, number of patients, SD, Standard Deviation; P, Significance (p-value set <0.05*).

Supplemental Digital Content 3.

Table S3. Patient-reported outcome measures in the IPC- vs plaster cast groups at 12 months

**ATRS 12 months**	**IPC**			**Plaster cast**			
	n	M	SD	n	M	SD	p
Strength	57	6.79	2.52	69	7.23	2.28	0.304
Tiredness	57	7.44	2.30	69	7.83	2.29	0.347
Stiffness	57	7.23	2.38	69	7.19	2.55	0.929
Pain	57	8.95	1.74	69	8.63	2.14	0.371
ADL	56	8.57	2.02	69	8.68	1.92	0.757
Uneven surface	56	8.46	1.92	69	8.83	1.79	0.278
Stairs/hills	56	8.30	1.83	69	8.45	1.95	0.670
When running	54	6.93	2.71	67	7.18	2.91	0.625
When jumping	55	6.24	2.99	68	6.57	3.09	0.542
Physical work	56	8.05	2.27	69	8.22	2.31	0.691
**Total sum**	53	77.8	17.2	68	78	19.3	0.962
**FAOS**							
Pain	55	94.75	8.24	68	94.0	9.89	0.671
Symptom	55	87.40	12.06	68	86.2	13.49	0.601
ADL	55	97.18	4.48	68	95.8	7.69	0.236
Sport and recreation	55	83.89	16.95	66	80.0	19.3	0.246
Foot and ankle-QoL	55	68.30	19.66	69	69.2	21.6	0.807
**PAS**							
Degree of activity	53	4.57	0.77	67	4.64	0.88	0.623
Degree of activity	53	3.74	1.15	63	3.98	1.10	0.237
**EQ5D index**							
EQ5D index	55	0.92	0.11	60	0.91	0.11	0.662

ATRS, Achilles Tendon Ruptured Score; The ATRS consists of 10 questions that can be scored from 0 to 10, leading to a total summed score of 0-100; a lower score indicates more symptoms and greater limitation of physical activity and quality of life; ADL, Activity of daily living; FAOS, Foot and Ankle Outcome Score consists of 5 subscales and where a total score of 100 indicates no symptoms and 0 indicates extreme symptoms; For PAS a score of 1 means that a patient is mostly sedentary, whereas a score of 6 means that a patient has engaged in heavy physical exercise several times per week. An EQ-VAS score of 0 is considered to be the worst imaginable health state and a score of 100 the superlative imaginable health state; n= number of patients, SD, Standard Deviation; P= Significance (p-value set <0.05*).

Supplemental Digital Content 4.

Table S4. Functional outcome in the IPC- vs plaster cast groups (12 months)

**Functional Outcome**		**IPC**			**Plaster cast**			
		n	M	SD	n	Mean	SD	p
Concentric power (W)	Injured	55	37.0	11.3	70	36.8	11.6	0.933
	Uninjured	56	44.8	9.22	70	45.8	11.9	0.608
	LSI (%)	52	74.2	32.3	69	81.3	24.3	0.170
Total conc. work (J)	Injured	55	1748	663	70	1690	716	0.645
	Uninjured	56	2526	761	70	2582	768	0.684
	LSI (%)	55	69.4	20.1	70	66.8	22.2	0.498
Heel-rise repetitions	Injured	55	24.7	7.42	70	24.4	7.98	0.841
	Uninjured	56	29.9	8.43	70	30.4	8.84	0.741
	LSI (%)	55	84.1	19.1	70	83.0	22.5	0.777
Avg. heel-rise height (cm)	Injured	55	8.28	2.18	70	8.36	2.23	0.852
	Uninjured	56	10.1	1.71	70	10.6	2.07	0.110
	LSI (%)	55	82.5	17.0	70	78.5	14.6	0.163
Eccentric power (W)	Injured	31	34.1	11.6	39	33.4	10.5	0.784
	Uninjured	32	42	9.18	39	42.9	11.1	0.705
	LSI (%)	32	82.6	22.9	39	78.2	15.8	0.340

Limb Symmetry Index (LSI = (injured/uninjured)*100) was calculated for concentric power, total concentric work, number of heel-rise repetitions, average heel-rise height and eccentric power.
